# Integrating haplotype-specific linkage maps in tetraploid species using SNP markers

**DOI:** 10.1007/s00122-016-2768-1

**Published:** 2016-08-25

**Authors:** Peter M. Bourke, Roeland E. Voorrips, Twan Kranenburg, Johannes Jansen, Richard G. F. Visser, Chris Maliepaard

**Affiliations:** 1 Wageningen UR Plant Breeding, Wageningen University and Research Centre, Droevendaalsesteeg 1, P.O. Box 386, 6708 PB Wageningen, The Netherlands; 2 Biometris, Wageningen University and Research Centre, Droevendaalsesteeg 1, 6708 PB Wageningen, The Netherlands

## Abstract

*****Key message***:**

**Linkage mapping can help unravel the complexities of polyploid genomes. Here, we integrate haplotype-specific linkage maps in autotetraploid potato and explore the possibilities for mapping in other polyploid species.**

**Abstract:**

High-density linkage mapping in autopolyploid species has become possible in recent years given the increasing number of molecular markers now available through modern genotyping platforms. Such maps along with larger experimental populations are needed before we can obtain sufficient accuracy to make marker-trait association studies useful in practice. Here, we describe a method to create genetic linkage maps for an autotetraploid species with large numbers of markers and apply it to an F1 population of tetraploid potato (*Solanum tuberosum* L.) of 235 individuals genotyped using a 20K SNP array. SNP intensity values were converted to allele dosages after which we calculated pairwise maximum likelihood estimates of recombination frequencies between all marker segregation types under the assumption of random bivalent pairing. These estimates were used in the clustering of markers into linkage groups and their subsequent ordering into 96 homologue maps. The homologue maps were integrated per chromosome, resulting in a total map length of 1061 cM from 6910 markers covering all 12 potato chromosomes. We examined the questions of marker phasing and binning and propose optimal strategies for both. We also investigated the effect of quadrivalent formation and preferential pairing on recombination frequency estimation and marker phasing, which is of great relevance not only for potato but also for genetic studies in other tetraploid species for which the meiotic pairing behaviour is less well understood.

**Electronic supplementary material:**

The online version of this article (doi:10.1007/s00122-016-2768-1) contains supplementary material, which is available to authorized users.

## Introduction

Polyploid species, where the basic diploid number of chromosome copies is exceeded, are increasingly becoming the subject of studies that aim to determine the links between genetic polymorphisms and phenotypic traits. To do this, researchers have needed to create maps of these species through linkage studies or sequencing efforts (or both). Affordable, high-throughput genotyping technologies together with greater computing power and the software needed to assemble these maps are enhancing our ability to perform such studies.

There have been a relatively large number of published tetraploid linkage maps in economically important allotetraploid species, such as cotton (*Gossypium hirsutum* L.) and durum wheat (*Triticum durum* L.). In contrast, autotetraploid maps are far fewer, with the exception of alfalfa (*Medicago sativa* L.) (Brouwer and Osborn 1999; Robins et al. 2008), potato (*Solanum tuberosum* L.) (Hackett et al. 2013; Meyer et al. 1998), and rose (*Rosa hybrida*) (Koning-Boucoiran et al. 2012; Rajapakse et al. 2001).

Methods for estimating marker dosage using (for example) SNP array data [e.g., fitTetra (Voorrips et al. 2011) or SuperMASSA (Serang et al. 2012)] have enabled researchers to exploit marker dosage information to generate polyploid linkage maps with a much higher marker density than before. Given the abundance of such marker sets, many polyploid maps continue to rely on 1:1 segregating markers, for which the coupling-phase recombination frequency estimates are identical to those for diploid species (Bertioli et al. 2014; Bourke et al. 2015; Vigna et al. 2016; Yu et al. 2015) (repulsion-phase estimates are not the same between species showing disomic inheritance, such as diploids, and those with polysomic inheritance, such as autotetraploids). However, there are many more marker segregation types that can be considered which may provide greater genome coverage as well as providing links between parental maps, important for subsequent analyses. In a tetraploid cross genotyped with bi-allelic markers for which dosage scores are available (assuming an absence of null alleles), there are nine fundamental marker segregation types: simplex × nulliplex (S × N), nulliplex × simplex (N × S), duplex × nulliplex (D × N), nulliplex × duplex (N × D), simplex × simplex (S × S), simplex × triplex (S × T), duplex × simplex (D × S), simplex × duplex (S × D), and duplex × duplex (D × D), according to the marker dosages carried by both parents. All other marker segregation types can be converted to one of these categories (Supplementary Table S1). These nine fundamental types have also been identified in previous studies, e.g., Hackett et al. (2013). Recently, methods to incorporate all marker segregation types from a tetraploid cross have been developed (Hackett et al. 2013). However, these methods do not automatically generate homologue maps, as these must be derived using chromosomal maps and phase information (Hackett et al. 2013; Massa et al. 2015). Here, ‘phase’ or ‘phasing’ means determining whether linked markers are physically on the same homologous chromosome within a parent. In our approach, we first develop separate maps for every parental homologous chromosome (termed ‘homologue’ here) using all marker segregation types, integrating them afterwards into one chromosomal map for each set of eight homologues. Marker phasing is thus an essential aspect of our approach, which is of importance in the development of marker haplotypes consisting of more than a single SNP marker.

Although methods to include double reduction in a linkage analysis have already been developed [either using two-point estimation (Luo et al. 2006) or multi-point estimation (Leach et al. 2010)], linkage analysis can be considerably simplified in autopolyploid mapping populations if it is assumed that only random bivalent pairing occurs. A review of metaphase I of autopolyploid meiosis found that bivalents accounted for approximately 70 % of the pairing structures observed (Ramsey and Schemske 2002) with quadrivalents accounting for approximately 29 % (there were a relatively few univalents or trivalents observed; more complex multivalents were not recorded at higher ploidy levels). Comparable rates have also been reported for potato (Bourke et al. 2015; Swaminathan and Howard 1953). In the computations of this study, we have assumed a complete absence of preferential pairing and multivalent formation. For example, in the case of S×N markers, a duplex score in the offspring would effectively be treated as a missing value, as it is not an expected score according to our model. However, we took care to examine what effect both preferential pairing and multivalents might have on the pairwise estimation of recombination frequency as well as the effects on the accuracy of marker phasing.

Although broadly similar, our mapping approach differs from that of Hackett et al. (2013) in the following respects:

1. The initial clustering of all marker segregation types into linkage groups is defined by their linkage to S × N or N × S (1:1 segregating) markers, enabling automatic marker phasing during mapping.
2. Homologue maps are first created (per parent) and then integrated into a single consensus map per chromosome using linear programming.
3. We include the results of a comparison study between two different methods for deciding the most likely phase.
4. Criteria are determined for binning markers together before map ordering.
5. All marker segregation types are included in the mapping (in particular, we also include S × T and T × S marker types).

In this study, we describe a method to perform linkage mapping in an autotetraploid species under the assumption of random bivalent pairing, and apply it to a genotyped mapping population of tetraploid potato. We explore some of the potential complications involved in polyploid mapping and discuss the implication of these for future mapping efforts.

## Materials and methods

### Plant material and genotyping

An F1 population of 237 individuals from the cross between two outbred tetraploid cultivars ‘*Altus*’ (parent 1 or P1) and ‘*Columba*’ (P2) was genotyped using the SolSTW Infinium SNP array which assays 17,987 SNPs (Vos et al. 2015). Markers were assigned dosages using the fitTetra package (Voorrips et al. 2011) as previously described (Bourke et al. 2015). Highly skewed markers (using a *χ* ^2^ test with *p* < 0.001) as well as markers with more than 10 % missing values were removed from the data set. In a previous study, a pair of duplicate offspring individuals was identified in this population as well as an individual which showed unrealistic numbers of recombinations (Bourke et al. 2015). The suspect individual was removed as well as the duplicate with most missing values, leaving a mapping population size of *N* = 235 individuals.

### Marker dosage conversion

A small number of markers for which one of the parental dosages was missing were examined and the likely parental dosage imputed using the observed offspring segregation (if possible), after which marker dosages were converted to their most fundamental form. In a tetraploid species genotyped using bi-allelic markers, the possible marker dosages classes are 0 (nulliplex), 1 (simplex), 2 (duplex), 3 (triplex) or 4 (quadruplex) depending on the number of copies of the ‘reference’ allele carried by that individual. Marker conversion simplifies the analysis by reducing the number of marker types that need to be considered. For example, simplex × nulliplex, triplex × quadruplex, triplex × nulliplex, and simplex × quadruplex markers all segregate in a 1:1 fashion and all carry a segregating allele inherited from parent 1 (P1). They can, therefore, be recoded as S×N markers using suitable score conversions in the offspring (Supplementary Table S1). Ultimately, this results in nine fundamental marker segregation types as previously mentioned. In determining linkage between S × T markers and other markers in P2, the set of S × T markers were recoded by symmetry into T × S to facilitate the calculations. The physical distribution of the segregating markers was visualised in MapChart 2.3 (Voorrips 2002) using the previously published centromere boundaries (Sharma et al. 2013).

### Linkage analysis

The maximum likelihood framework for determining pairwise estimators for recombination frequency (*r*) and their significance (LOD) scores under the assumption of random bivalent pairing has already been described in Hackett et al. (2013). We independently derived the likelihood functions for all possible marker pairs and phases (of which we counted 92 possible combinations between the nine fundamental marker types mentioned) using routines written in Mathematica 10.0 (Wolfram Research Inc. 2014). We describe the procedure through a worked example in Appendix 1 (Supplementary File S1). The maximum likelihood functions (for each of these 92 cases) were coded in R (R Core Team 2016) for use in the linkage analysis.

### Marker phasing

To explain the concept of phasing by way of example, there are three possible phases between a D × N and a D × S marker: ‘coupling’, ‘mixed’, and ‘repulsion’, with ‘mixed’ implying only one pair of duplex alleles is in coupling phase in P1 (there is no phase consideration in P2, since one of the markers is nulliplex in that parent). For pairs of markers with segregating alleles from both parents, such phases are combined (e.g., ‘coupling mixed’ refers to coupling phase in P1 and mixed phase in P2). One criterion for selecting the correct phase (and hence the correct estimator for *r*) is to use the maximum of the log-likelihood function between phases for which *r* ≤ 0.5 (Hackett et al. 2013) which we refer to as MLL. Another possibility is to choose the minimum estimate of *r* over all phases (which we term MINR). We performed a simulation study to determine which of these criteria was optimal across all possible marker pair combinations using the simulation software PedigreeSim (Voorrips and Maliepaard 2012). One hundred separate populations were generated for each of three population sizes (F1 = 100, 200 and 400). Each simulated individual carried a single chromosome with 100 marker positions spaced 1 cM apart. All possible marker types were assigned to each of these loci, with a random assignment of the segregating alleles across homologues. For each simulated population, phasing accuracy was determined by recording the proportions of correctly phased pairs using both the MLL and MINR phasing strategies. In a few cases, it was not possible to distinguish between phases (e.g., S × S with D × D ‘coupling-repulsion’ and ‘repulsion-coupling’ phases produce precisely the same *r* estimates and LOD scores). In diploid species, an analogous situation can arise in cross-pollinated species where certain marker type combinations cannot be phased (e.g., AB  ×  AB with AB × BA), in which case other marker segregation types are needed to complete the phasing (Maliepaard et al. 1997). We dealt with such instances by considering both phases to be equally correct (since we do not use these particular phase assignments themselves, only their *r* and LOD values).

### Linkage group identification and marker clustering

Preliminary identification of linkage groups was performed by clustering the S × N (and N × S) markers, based on the LOD of the recombination frequency estimate between marker pairs. A routine for marker clustering was written in R using a grouping algorithm analogous to that employed by the JoinMap software (Stam 1993; Van Ooijen 2006; Van Ooijen and Jansen 2013). Of the 1497 N × S markers (Table 1), three did not have any strong linkage to other N × S markers and were, therefore, removed at this stage (we were later able to ‘rescue’ one of them when more markers were assigned to chromosomes). At a clustering threshold of LOD 4, the N × S marker data divided into 12 clusters. We visualised how clusters split across different LOD values for these 12 putative chromosomes in R, allowing the identification of tightly linked subclusters (putative homologues or fragments thereof). In the majority of cases, these large clusters split into four subclusters at higher LOD values (as expected for a tetrasomic species). However, one cluster (of 15 markers) could not be further subdivided at higher LOD values. Therefore, we assumed that this cluster represented (part of) one homologue and used the repulsion linkage information to assign it to one of the other clusters. Another cluster broke down into two clear subclusters at LOD 5 (i.e., it contained two chromosomal groups), which further subdivided into 9 subclusters at LOD 6. Clustering in P1 was more straightforward, with 12 clear chromosomal clusters emerging at LOD 4.

**Table 1:** Tetraploid marker segregation types after filtering (adapted from Bourke et al. 2015)

Parental dosage	Segregation	Amount^a^
Simplex × nulliplex	1:1	1690
Nulliplex × simplex	1:1	1497
Duplex × nulliplex	1:4:1	409
Nulliplex × duplex	1:4:1	442
Simplex × simplex	1:2:1	924
Simplex × triplex	1:2:1	410
Duplex × simplex	1:5:5:1	596
Simplex × duplex	1:5:5:1	665
Duplex × duplex	1:8:18:8:1	279
Total		6912

^a^Number of SNP markers after marker conversions have been made

Cluster numbers were replaced with chromosome numbering for consistency with the reference physical map using marker positions given by (Vos et al. 2015). In P1, chromosomes 4 and 10 contained five subclusters with the rest having four. In P2, chromosomes 3, 5, and 9 were found to contain five subclusters at this stage, the rest having four. In cases where more than four subclusters are identified, a visualisation of cross-cluster phase assignments allowed us to quickly identify which subclusters were (albeit distantly) linked in coupling phase, resolving the S × N and N × S marker data into 12  ×  4 × 2 = 96 linkage groups, the expected number of homologues. Following this, the vast majority of markers within the complete data set were unambiguously assigned to homologue clusters using coupling-phase linkage with S × N markers (a single linkage above an LOD threshold of 3 was used as evidence of linkage, although in practice there were often hundreds of such linkages identified). S × N markers are extremely useful for this step as they unambiguously tag a single homologue. Where multiple assignments were possible, assignments with the greatest number of significant coupling linkages (LOD > 3) were chosen as the most likely linkage groups. For those markers which could not be completely assigned to the expected number of homologues in both parents due to poor linkage with S × N markers, linkage analysis was performed between these markers and all other marker segregation types to identify their most likely chromosome and homologue assignment. Finally, the marker data were split into 12 subsets (one for each chromosome) and a complete pairwise linkage analysis was run between all markers within each chromosome.

### Map construction

#### Homologue mapping

Per chromosome, there are eight homologues that can be mapped separately in an autotetraploid. Markers which appear on these homologues are already identified through their linkage with S × N (or N × S) markers. In addition to the coupling-phase linkages considered, we also included some repulsion-phase linkages in our homologue maps. Markers with at least one duplex allele are completely symmetrical between the ‘reference’ and ‘alternative’ alleles in the duplex parent. For example, D × N markers are initially assigned to two homologues, these being the homologues on which the reference allele can be found. However, it is equally informative to consider the pair of ‘alternative’ alleles from the same parent, as these carry the same linkage information as the reference alleles. Therefore, we used D × N markers in the mapping of four homologues, S × D and D × S for the mapping of five homologues and D × D markers for all eight. All of these marker types (as well as S × S and S × T) are extremely useful as “bridging” markers for the integration of homologue maps.

There remained some linkages that we did not exploit, e.g., S × N and S × N in repulsion. The variance of these repulsion-phase estimates is high (and hence, LOD values are low), and, therefore, the added computation time from including these estimates is not worth the marginal increase in linkage information that they yield (a similar conclusion was arrived at in previous studies, e.g., Ripol et al. 1999).

#### Marker binning

Linkage information per homologue was first assembled into two pairwise matrices (one for *r* estimates and one for LOD scores), after which the strength of linkage was tested to determine whether marker binning was possible—i.e., markers with a small recombination frequency estimate (*r*) of low variance (thus high LOD) were binned together. The minimum (non-zero) number of recombinations that can be observed in a mapping population of size *N* (and hence 2*N* gametes) is one, in which case the smallest non-zero *r* estimate should be 1/(2*N*) (ignoring the influence of errors). Given an average missing value rate of *µ*, a population size adjusted for missing values is approximately *N*_*a*_ = (1 − *μ*)*N*, and therefore, *r*_min_ ≈ 1/(2(1 − *μ*)*N*). Estimates of *r* that were smaller than this value were taken as being below the threshold of minimum resolution (*r*_min_).

Not all estimates of the recombination frequency are equally accurate. Therefore, we determined criteria for binning markers together with a high degree of confidence. To achieve this, we ran simulations using PedigreeSim (Voorrips and Maliepaard 2012) and recorded the LOD scores as well as the range of true recombination frequencies for *r* estimates below the threshold of minimum resolution over a wide range of population sizes (F1 = 100–1000 in steps of 100) and rates of missing values (0–20 % in steps of 5 %). We chose a maximum allowable deviation between the true and estimated *r* as 0.01 (approximately 1 cM) and determined the corresponding LOD score to ensure this over all possible marker pair combinations (hence, we took the most stringent LOD threshold to cover all cases). For each population size and rate of missing values, we examined the distribution of LOD scores for those recombination frequency estimates for which the deviation was less than 0.01 (Supplementary Figure 1.b). From this, we could determine a suitable LOD threshold for marker binning as a function of mapping population size and rate of missing data.

Given a set of markers that have been binned together, we chose the S × N marker with the fewest number of missing values for mapping (S × N recombination frequency estimates with other marker types are exact as opposed to being numerically approximated); in bins with no S × N markers, the marker with the fewest missing values was selected.

#### Marker ordering

The remaining marker data (after binning) were converted into pairwise-data file format (.pwd) for each homologue and imported into JoinMap 4.1 (Van Ooijen 2006) for ordering. Three rounds of mapping using the weighted least squares algorithm were used (using the default settings with a “jump threshold” of 5), and with Haldane’s mapping function used for distance conversion. Map files were subsequently exported, and all binned markers were readded to the maps.

#### Map integration

**Fig. 1:**
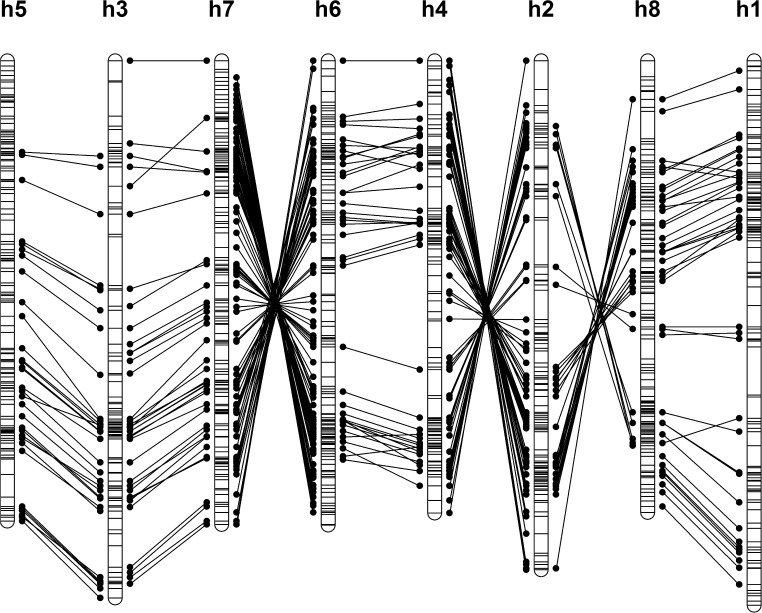
Visualisation of map connections on chromosome 7. *Note* In this example, there were sufficient bridging markers to provide connections between all homologues. Three reversals were needed to ensure that all homologues were consistently orientated before map integration

The homologue maps were first reorientated (if necessary) before integrating. Map reorientation was achieved by locating bridging markers between the maps and determining the correlation between the cM positions of these markers. A negative correlation suggests that maps are orientated in reverse order relative to one another. Since not all homologues necessarily share bridging markers, the R package igraph (Csardi and Nepusz 2006) was used to find an order of comparison through the eight homologue maps per chromosome to allow stepwise correlations to be calculated (for example, 5-3-7-6-4-2-8-1 might be one such order). An example of this is shown in Fig. 1 for chromosome 7. In this example, three separate reorientations were required to ensure consistency in orientation. When all eight maps were similarly orientated, the R package LPmerge (Endelman and Plomion 2014) was used to integrate them. LPmerge eliminates the minimum number of constraints in the marker order of the underlying maps (conflicts in order between maps) to generate what is called a “feasible system,” and then uses linear programming to find the solution with the minimum error between the underlying maps and the integrated map (Endelman and Plomion 2014).

### Map quality checks

We checked the quality of our linkage maps using three different approaches:

- consistency between the integrated maps and the underlying homologue maps;
- comparison with the reported physical position of the mapped markers;
- comparison to other published tetraploid potato maps.

#### Consistency between the integrated maps and the underlying haplotype-specific homologue maps

We compared the marker positions on the underlying homologue maps and the integrated maps to identify possible map distortion. Map distortion is partly reflected in the absolute error (*δ*) between the underlying maps and the integrated maps. However, in cases where the telomeres of all homologues are not equally covered by markers, shifting the 0-cM position may occur to align the maps, contributing to an apparent increase in *δ* without implying any map distortion. We ran a simple linear regression between the integrated map positions (cM) and the underlying homologue positions (cM) and recorded the slope and adjusted *R* ^2^ values of the fit as well as visually inspecting each chromosome to identify potential distortion.

#### Comparison with the physical position of the mapped markers

The physical positions of the markers were taken as described in Vos et al. (2015). Plotting the genetic positions against the physical positions allowed us to identify whether our maps were correctly orientated (i.e., 0 cM corresponding to the lowest bp value) and we reorientated our maps if necessary. In cases where a discrepancy was found between the reported chromosome and that found through our linkage analysis, we BLASTed the marker EST sequences (provided in the Supplementary Material of Vos et al. (2015) and reproduced here) to the potato DM1-3 Pseudomolecules reference genome version 4.03 (Hirsch et al. 2014) to check the marker positions (website: http://solanaceae.plantbiology.msu.edu/blast.shtml, accessed 16.11.2015).

#### Comparison to other published tetraploid potato maps

The SolCAP 8303 Infinium array (Felcher et al. 2012) has been used to genotype at least two other published tetraploid potato mapping populations (Hackett et al. 2013; Massa et al. 2015). We compared the genetic map positions of the common markers as a further check on the validity of our maps.

### Simulation study to check mapping assumptions

Two crucial assumptions were made prior to mapping: that there is no preferential pairing behaviour between any homologues and that all pairing is between bivalents (as opposed to trivalents or quadrivalents), i.e., there is no double reduction. It had previously been established that the rate of quadrivalent pairing in this population was between 20 and 30 % (Bourke et al. 2015). In contrast to previous studies which also rely on these assumptions, we wanted to test what effect deviations from these assumptions might have on our ability to produce unbiased and accurate estimates for *r* between marker pairs as well as the effects on phasing accuracy.

We simulated mapping populations using different degrees of quadrivalents and preferential pairing in PedigreeSim (Voorrips and Maliepaard 2012). The simulation parameters we chose were: population sizes of 100, 200, and 400 F1 offspring, levels of preferential pairing from 0 (fully random pairing or tetrasomic behaviour) to 1 (fully preferential pairing or disomic behaviour, associated with allopolyploidy) in steps of 0.1, or fraction quadrivalents from 0 to 1 in steps of 0.1. For each population size, we generated 100 separate populations. Each simulated individual carried a single chromosome with 100 marker positions spaced 1 cM apart. All possible marker types were assigned to each of these loci, with a random assignment of the segregating alleles to homologues at all marker positions. In total, we simulated 6600 populations to cover our chosen range of quadrivalents and preferential pairing for these three population sizes and number of repetitions ((11 + 11) × 3 × 100).

After the populations were generated (i.e., their SNP dosage genotypes known), we ran our linkage analysis functions across all populations. Given that the data sets were simulated, the true recombination frequency and correct phasing between all marker positions was known, allowing us to test whether our estimation of recombination frequency and marker phasing was robust against these deviations from the random bivalent model. To generate average results from the 100 repeat populations per setting, we ran a simple linear regression on the estimated *r* versus true *r* values, recording the slope, intercept, *R*_adj_^2^ and residual standard deviation of the regression. We also recorded the proportion of situations not estimated (e.g., due to undefined numbers in the likelihood equation) and the proportion of pairing situations that were correctly phased.

## Results

### Genotypes

**Fig. 2:**
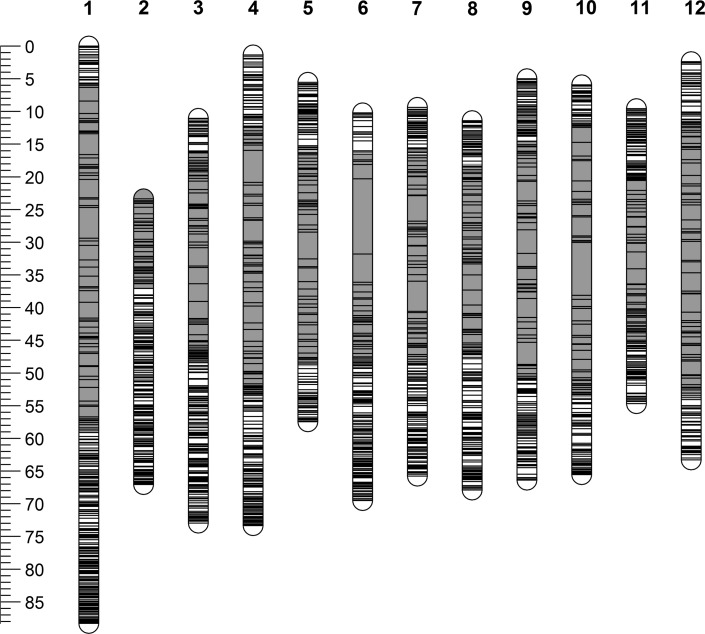
Distribution of 6836 of the 6912 segregating markers used in this study for which a physical assignment was available. *Note* Distances shown in Mbp. *Yellow* regions indicate centromeres, as previously defined (Sharma et al. 2013) (colour figure online)

The number of SNP markers that were available for mapping after marker filtering and quality checks was 6912, as outlined in Table 2. The breakdown of marker segregation types after marker conversions were performed is provided in Table 1. Approximately 46 % of the markers segregate in a 1:1 fashion and these were mapped in a previous study (Bourke et al. 2015). The physical distribution of all marker types for which physical positions were available is given in Fig. 2, highlighting the difference in marker distribution between telo- and centromeric regions.

**Table 2:** Breakdown of SNP marker numbers after quality filtering (adapted from Bourke et al. 2015)

Steps in SNP filtering	Amount	%
SolSTW Infinium array total # SNPs	17,987	100.0
Dosages assigned by fitTetra^a^	15,266	84.9
F1 pattern acceptable^b^	13,774	76.6
Monomorphic	6558	36.5
Polymorphic	7216	40.1
Polymorphic and ≤10 % NA values	6912	38.4

^a^Markers not scored were monomorphic or not clearly resolved. Markers with a single missing parental dosage score which was imputed have been included

^b^Criteria for lack of F1 fit: presence of null alleles, >3 % invalid scores, highly skewed segregation (*p* < 0.001)

### Linkage analysis and marker clustering

#### Maximum likelihood pairwise estimates for *r*

We counted 92 separate marker type and phase combinations for which we derived maximum likelihood functions, although this may contain scenarios that can be counted together [previously, 67 situations have been reported (Hackett et al. 2013)]. For clarity, we provide a table of all the possible marker type and phase combinations we considered (Supplementary Table S2). In many cases, the maximum likelihood equation cannot be solved analytically, in which case we used Brent’s algorithm (Brent 1973) to numerically estimate the recombination frequency with the highest likelihood, constrained to the interval [0, 0.5]. It is also possible that a negative estimate for *r* could be the most likely (this can occur in low-information situations, involving repulsion phases). We examined the true values of *r* underlying such cases using simulated data and found that a wide range of true *r* values were possible. Whenever *r* < 0 was found, we artificially set *r* = 0.499, LOD = 0 and phase “unknown,” thereby excluding these estimates from the map ordering step.

#### Optimal phasing strategy

Our simulations revealed the optimum phasing strategy to use for different marker combinations (Supplementary Figure 2). The maximum log likelihood strategy (MLL) as proposed by Hackett et al. (2013) proved in general to be a very good method of selecting the correct phase (and hence the correct estimate for *r*). In only one situation did we find MINR to outperform MLL, namely, S × S with S × S markers. The improvement was, however, marginal: at a population size of 200 individuals for example, MINR gave 93.8 % accuracy versus 90.9 % accuracy using MLL. Whenever phase was incorrectly assigned, we found that the LOD score was also low (and the recombination frequency estimates tended to be high)—in other words, incorrect phasing only occurred in poorly informative situations which would have little or no impact on the subsequent ordering step (especially since our mapping strategy favours coupling-phase estimates which tend to be more informative than those of repulsion-phase). In all situations involving at least one S × N marker, there was no difference between the two methods. For the case of S × S paired with S × T markers, the accuracy of MINR appeared at first glance to be higher. However, this particular combination of markers contains an essentially unestimable phase with extremely high variance (repulsion/coupling phase; see also Results section “Simplex  ×  triplex markers”). Removing this phase from the accuracy calculation, MLL was found to perform significantly better among the phases that actually *matter* in this combination. Finally, phasing accuracy was found to slightly increase as a function of population size, with 92 % accuracy on average for a mapping population of size 400 (compared with 89 % accuracy for a population of 200, and 84 % for a population of 100). A breakdown of the phasing accuracy rates is provided in Supplementary File S2.

### Map construction and integration

#### Simplex × triplex markers

S × T markers have previously been reported as problematic [where they are termed XSS markers (Hackett et al. 2013)]. When we examined the issue, we found that S × T in combination with S × S produce highly variable estimates for *r* in repulsion/coupling phase, where the estimates for *r* are essentially random. They are the only marker combination (and phase) that exhibit such behaviour. Therefore, we artificially set LOD = 0 in this phase (it would be small but non-zero otherwise) which automatically excludes these estimates from exerting any influence on map ordering.

#### Marker binning

**Fig. 3:**
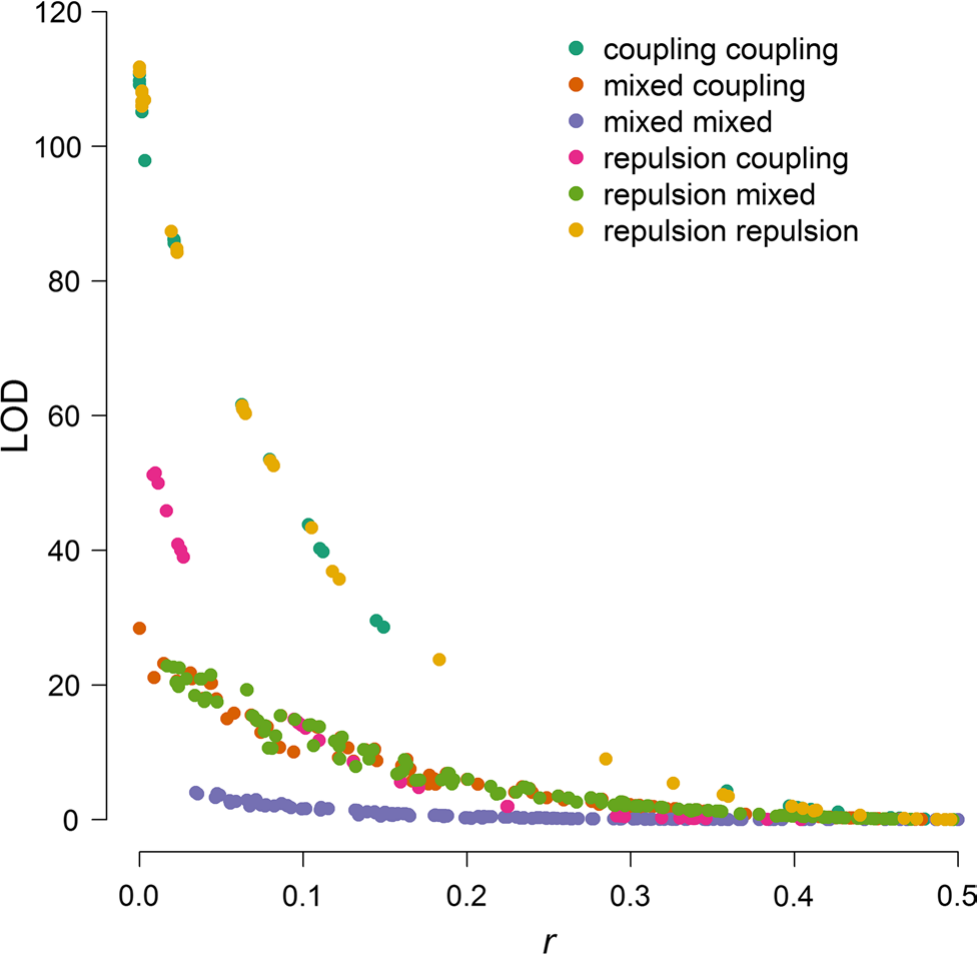
Estimated recombination frequencies (*r*) versus associated LOD for duplex × duplex marker pairs on potato chromosome 1. *Note* Only 6 of the 9 possible phases were identified for marker pairs on this chromosome

Once all linkages between markers had been estimated, we were in a position to identify cosegregating markers. The *r* and LOD estimates give a convenient measure of linkage which can be applied across all marker segregation types in a binning procedure. An example of the relationship between *r* and LOD for pairs of D × D markers is visualised in Fig. 3. As higher LOD values correspond to a lower standard error in *r*, we wanted to define thresholds for *r* and LOD which would identify markers which cosegregate with a high degree of confidence.

We previously introduced the concept of the threshold of minimum resolution for recombination frequency, *r*_min_. Given our mapping population size and missing error rate (in the filtered data set), we estimated *r*_min_ to be approximately 0.0022—the smallest non-zero recombination frequency we should be able to observe. From our simulation of different population sizes and rates of missing data, we observed a clearly linear relationship between the mapping population size and the LOD threshold needed to ensure a margin of error of less than 0.01 in the estimation of *r* (Supplementary Figure 1.c). By performing a linear regression between the LOD thresholds and the adjusted mapping population sizes (*N*_*a*_), we were able to derive an empirical relationship between a binning LOD threshold and the adjusted population size, which ensures that this margin of error in *r* is not exceeded:

$$ {\text{LOD}} \approx 23.43 + 0.1158N_{a} $$

Given our data set, we binned markers together if we found that the pairwise *r* estimate was less than 0.0022 and the LOD for that estimate exceeded 50.4. In total, 10,649 markers were used in the map ordering step across 96 separate homologue maps (note that some markers were present multiple times, because they are present on multiple homologues), after which 7099 binned markers were reassigned a position to give a total of 17,748 map positions (Supplementary Table S3). As binning was performed using a nearest-neighbour clustering, there is a danger that binned markers might have a non-negligible distance between them. When we examined this, we found that the maximum recombination frequency estimate between binned markers was 0.031, or approximately 3.3 cM using Haldane’s mapping function (Supplementary File S5). However, the mean inter-marker distance within bins was only 0.12 cM, and almost 99 % of binned markers were less than 1 cM from each other. In other words, our binning strategy rarely appears to have falsely binned markers together. These 17,748 map positions represented 6910 unique marker loci, i.e., only two of the 6912 markers available for mapping were not mapped. We suspect that the two unmapped markers which showed no linkage may have harboured abnormally high numbers of errors, although we cannot verify this. A full list of all marker positions per chromosome is provided in Supplementary File S3.

#### Map integration

LPmerge (Endelman and Plomion 2014) was run for all 12 linkage groups to determine the integrated maps with lowest absolute error *δ* between the homologue maps and the integrated map per linkage group (referred to as RMSE by the authors). Although the LPmerge algorithm is deterministic (Endelman, personal communication), we found the resulting maps differed when the input maps were flipped. This suggests that the current version of LPmerge should be run twice to identify the best integrated map. The maximum interval size (see Endelman and Plomion (2014) for a description) was set at 8 (default 4), and the map with the minimum error was saved for the final selection of the “globally” optimal integrated maps. LPmerge currently reports the error associated with each possible solution but does not save this information automatically. Therefore, we altered part of the source code to create an output file of the errors and map lengths per maximum interval, allowing us to identify the best results. The altered source code of the LPmerge function is provided in Supplementary File S6.

### Map quality

#### Consistency between the integrated maps and the underlying haplotype-specific homologue maps

**Fig. 4:**
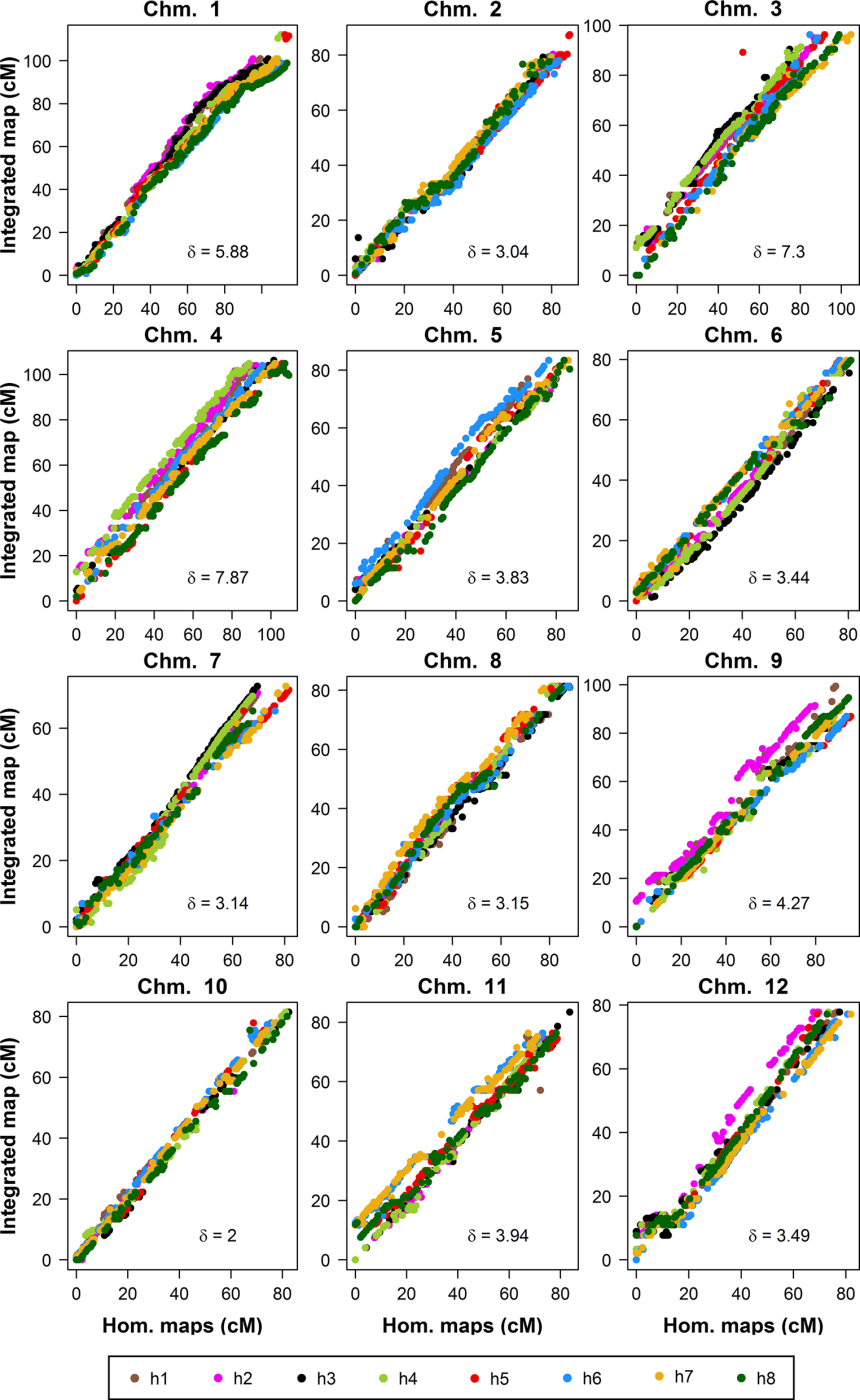
Comparison between marker positions on underlying homologue maps and integrated map positions for potato chromosomes 1–12. *Note Different colours* denote different homologues. *δ* denotes the absolute error between the eight homologue maps and the integrated map, as calculated by LPmerge (colour figure online)

We visualised the relationship between the homologue maps and the integrated map for each chromosome (Fig. 4). Apart from a small number of ‘kinks’, there was a very high level of linearity observed between the component maps and the integrated maps as well as an acceptable correspondence in map lengths (Supplementary Table S3), demonstrating that the integration step did not create undue distortion. This linearity was also reflected in the high *R*_adj_^2^ values associated with the regression analysis—with a minimum of 0.97 and a mean of 0.99 (i.e., essentially colinear). The slopes and adjusted correlation coefficients of the different maps are provided in Supplementary Table S4.

#### Comparison with the physical position of the mapped markers

**Fig. 5:**
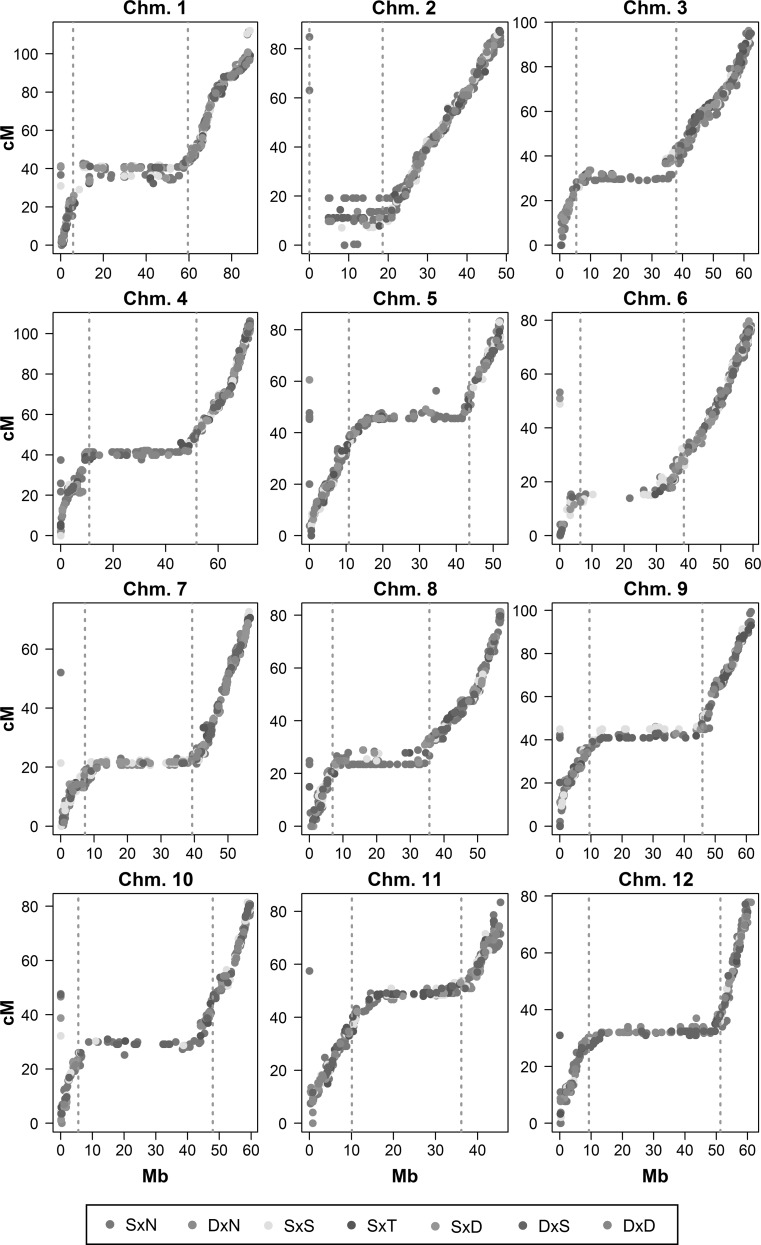
Comparison between physical and integrated genetic maps for 6872 mapped markers on potato chromosomes 1–12. *Note Different colours* denote different marker segregation types. Centromeres as defined in (Sharma et al. 2013) are shown with *dashed lines*. 38 markers for which the chromosome assignment differed were removed before plotting. Outlying markers positioned at 0 Mb had no physical position—for which we suggest approximate positions based on these plots (c.f. Supplementary File S4) (colour figure online)

One of the advantages of developing mapping theory and software using data from potato is the availability of physical maps which provide a reference marker position (Felcher et al. 2012; Potato Genome Sequencing Consortium 2011; Vos et al. 2015). Reorienting the integrated genetic maps if necessary, we found the expected profiles for all chromosomes (Fig. 5) and could also clearly identify markers for which the chromosome assignment on the physical map appears to be incorrect. The physical location of 68 other markers was previously unknown (recorded as 0 Mb on chromosome 0), for which we can now provide an approximate physical position based on these plots (Supplementary File S4). These plots also provided information on the location of the pericentromeric regions. We found differences between our identified pericentromeric boundaries and those previously reported for potato chromosomes 5, 6, 10, and 11 (Sharma et al. 2013). In all these cases, we noted that the published regions were too large, i.e., some stretches of the pericentromeric regions of these chromosomes show little or no suppression of recombination. One issue that did arise on chromosome 2 was the mapping of what we suspect is a centromeric marker to a non-centromeric position (Fig. 5). All markers binned with this marker were subsequently also at a non-centromeric position. When we ran the mapping without binning in this homologue, we saw essentially the same result—i.e., marker binning was not to blame. The integration of information from telocentric chromosomes may result in such minor ordering errors given that multi-point estimates (from which the map is ultimately derived) are one-sided at the telomeres.

#### Comparison to other published tetraploid potato maps

A subset of the SolSTW 20K SNPs came directly from the SolCAP 8303 SNP array, with 3684 of these having an acceptable F1 pattern after fitting, of which 2707 segregated. We mapped 2706 of these SolCAP markers, allowing a direct comparison of our genetic maps with previously published tetraploid maps which use these markers (Hackett et al. 2013; Massa et al. 2015). Only a single marker from this set was found to have been assigned to a different linkage group between the three studies (solcap_snp_c1_15085), which was mapped on chromosome 6 by Hackett et al. and which both we and Massa et al. mapped on chromosome 4. We double-checked the physical position by BLASTing the marker sequence against the potato genome (Hirsch et al. 2014) and found it produced a single hit on chromosome 4.

A comparison of the maps is shown in Supplementary Figure 3. In general, our map positions correspond well with those of previous studies, apart from chromosome 9 where we found that a group of markers most likely to be centromeric (based on a comparison with the physical map) were mapped at 110 cM by Hackett et al. (2013) even though the pericentromeric region of chromosome 9 is positioned at approximately 50 cM in their map. There also appear to be several cases where markers were binned together by Hackett et al. (2013) which we assigned to different genetic positions (Supplementary Figure 3, chromosomes 2, 4, 8, and 11). However, the binning strategy employed by Hackett et al. (2013) differs considerably from our approach, in that markers are not automatically binned before map ordering, but only end up in a “bin” if they fail to map after two rounds of JoinMap’s weighted regression ordering algorithm. Despite these discrepancies, the mapping performed in previous studies is broadly consistent with our results.

### Effect of quadrivalents and preferential pairing on mapping

#### Quadrivalents

One notable effect of quadrivalent pairing in meiosis is the phenomenon known as double reduction, where two copies of the same parental homologue segment are transmitted to an offspring. It has previously been shown that quadrivalents have a relatively minor impact on recombination frequency estimates of pairs of S × N markers (Bourke et al. 2015). Here, we extend the analysis to all possible marker segregation types of a tetraploid cross. In general, we can confirm our previous finding that quadrivalents have a minor impact on *r* estimates for most marker pairs and phases, but lead to an underestimation of *r* when the proportion of quadrivalents approaches one (e.g., S × D and D × D in coupling/repulsion phase, Supplementary Figure 4). However, no observations of such high proportions of quadrivalents have been reported yet (as far as we know) in an autopolyploid species (Bourke et al. 2015; Ramsey and Schemske 2002; Swaminathan and Howard 1953). In Supplementary File S7.a, we provide full details of the results of this study.

#### Preferential pairing

Preferential pairing constitutes a much greater deviation from the assumption of random bivalent pairing, and this was reflected in the results of the simulation study. We again saw a downward bias in *r* with greater levels of disomic behaviour, which was accompanied by a drop in the correlation between the true and estimated values. A higher population size can help to mitigate these effects, but when the rate of preferential pairing (*p*) exceeds ~0.7, this makes little difference. Correct phase estimation was surprisingly robust against preferential pairing, although, in a fully disomic situation (*p* = 1), it was not possible to estimate *r* in certain cases (specifically, the combination between a duplex and simplex allele in either parent when both alleles are present on the same bivalent, leads to on average 33 % inestimable values). Nevertheless, recombination frequency estimates showed high levels of stability and robustness, even when significant levels of preferential pairing occur. For identifying linkage groups, preferential pairing has almost no impact on the accuracy of coupling linkage estimates with S × N markers (which we use for marker clustering), with the possible exception of D × D markers. An example of the results for S × D and D × S markers in coupling/coupling phase is given in Supplementary Figure 5. In Supplementary File S7.b, full details of the results of this study can be found.

## Discussion

### Homologue mapping

In our approach, we identify all homologous linkage groups first and map them separately, combining them together in the final step using bridging markers (markers mapped on more than one homologue). There are a number of advantages to this, the first of which is the division of large computational tasks into parallel subtasks which results in a significant time-saving. Given that marker data sets are generally increasing in size, it is likely this approach will become increasingly necessary in future polyploid mapping studies. Marker phasing is performed automatically in the initial clustering step and does not have to be calculated afterwards. We also avoid potential map ordering issues by only using the most informative linkage information in map construction. In the case where we identified very high variance associated with *r* (S × S and S × T in coupling/repulsion phase), we excluded these estimates from the map ordering step by artificially setting LOD = 0.

The use of haplotypes has been shown to have greater statistical power than single-marker approaches in diploid association studies, particularly those involving humans (de Bakker et al. 2005). Our mapping method focuses on creating chromosome-length SNP haplotypes (homologue maps) and, therefore, could facilitate multi-SNP marker QTL studies rather than those based on single-marker positions. Having separate homologue maps will also enable the further exploration of QTL positions, allowing the identification of haplotypes responsible for the phenotypic variation observed. Integrating these homologue maps is a prerequisite for further QTL analyses that use inheritance probabilities instead of marker dosages as explanatory variables (Hackett et al. 2013, 2014).

Finally, in mapping populations where the meiotic behaviour is not consistent between parents, it is preferable to map each parent separately, given a framework that can incorporate, e.g., preferential pairing in the estimation of recombination frequency. Our work in other polyploids (particularly ornamental species) suggests that accommodating such meiotic differences is likely to become a regular feature of future mapping work. Parental mapping would also be needed in a tetraploid × diploid cross (for example) because of the different ploidy levels of the two parents.

### The necessity of simplex × nulliplex markers

One of the potential pitfalls of our mapping strategy is its reliance on S × N markers (both in terms of numbers and distribution). Without an abundance of this marker type, we would have to adapt our mapping approach. S × S markers can also be used to define homologous chromosomes, but with the added complication of dividing the marker data into 4  ×  4 = 16 cross-parental groupings rather than 4 + 4 = 8. However, it is feasible to use additional phasing information to determine from which parental allele the coupling-phase linkage originates. A viable alternative would be to adopt the mapping strategy described in (Hackett et al. 2013). Nevertheless, we have yet to encounter situations where the number of S × N (or N × S) markers would cause such a restriction—indeed, they tend to be the most abundant marker segregation type that we have encountered across multiple populations.

### Marker binning

**Fig. 6:**
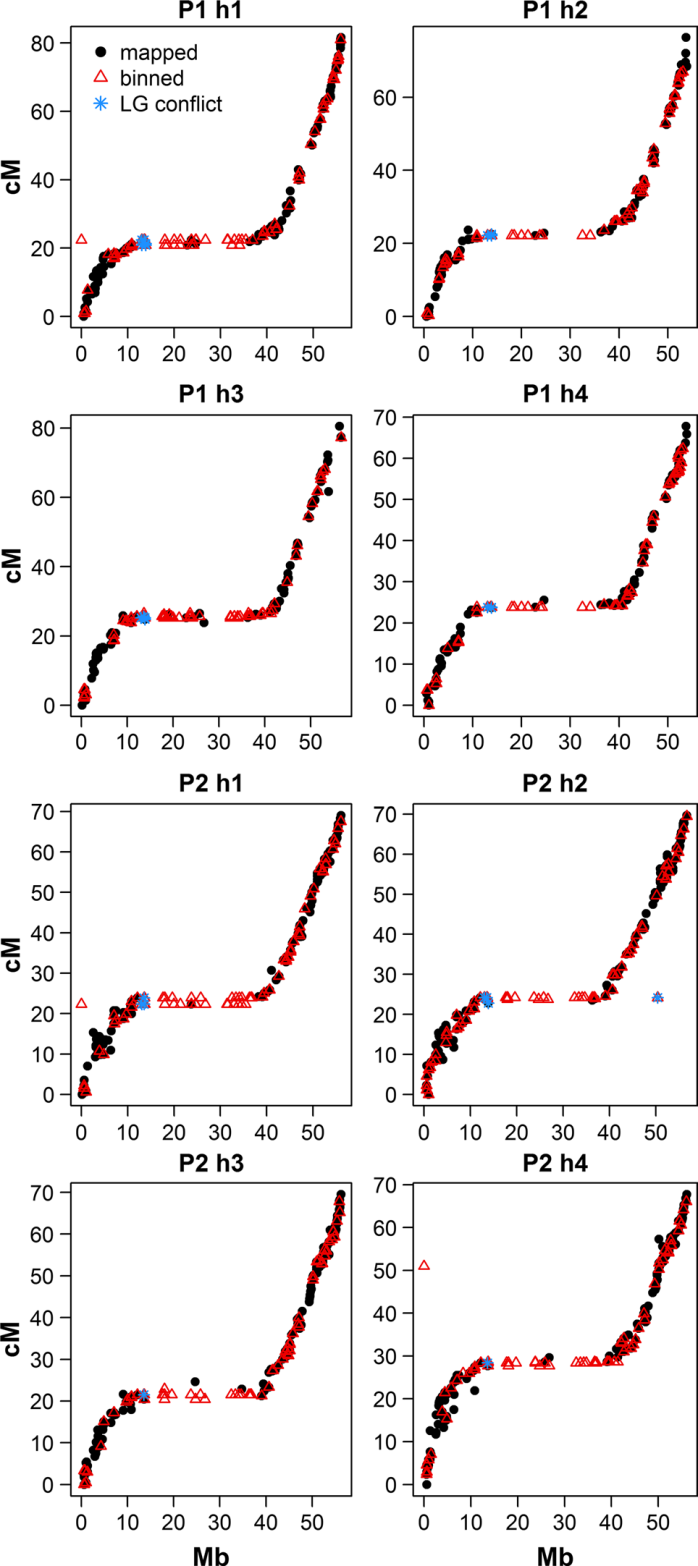
Distribution of binned markers on eight homologue maps of potato chromosome 7. *Note* Mapped markers (used in the marker ordering step) are shown as *black dots*, *binned markers* (removed prior to marker ordering) are shown as *red open triangles* and were added after mapping. LG conflict (*blue stars*) refers to markers for which the chromosome assignment on the physical and genetic maps differs (colour figure online)

Marker binning has an enormous impact on the speed of marker ordering, particularly since the timing of the weighted linear regression map ordering algorithm is at least quadratic with the number of markers used. Almost half the markers were binned during the mapping, reducing the effective number of marker loci from 6910 to 3980. This was also reflected at the homologue level, reducing the mean number of markers from 185 to 111 markers per homologue map. An examination of where these binned markers came from revealed that they were relatively well distributed, but were particularly abundant in the pericentromeric regions as one would expect (chromosome 7 is shown as an example in Fig. 6).

Despite the efficacy of marker binning, the trend continues to be towards even larger marker data sets. In cases where the marker set size becomes unworkably large, there are a number of simple amendments to our method that could be considered, for example:

1. Binning more markers to create sparse framework maps initially; further saturation for fine-mapping can be confined to interesting regions after initial QTL analyses.
2. Subdividing homologue marker clusters into smaller groups and mapping these segments separately before merging.

Nevertheless, the development of faster algorithms for marker ordering is a likely prerequisite for future mapping studies in polyploid species involving large population sizes (and more markers). For now, it appears that the weighted linear regression criterion of JoinMap remains the best option to produce accurate maps given pairwise recombination frequency estimates with variable information content.

### Map integration

One behaviour which we did not expect was the variability of LPmerge depending on the relative orientation of the input maps, which has not been described by the authors (Endelman 2011; Endelman and Plomion 2014), or in any subsequent publication known to us which uses this package. Higher numbers of bridging markers will probably improve the stability of the integrated map solution found between successive runs, although we recommend that the integration step be repeated over a range of maximum interval sizes and using both forward and reverse orientations to ensure that the best integrated map has been found.

### Application to other tetraploid species

The methods developed here can be directly applied to other tetraploid species. Our results show that mapping under the assumption of random bivalents is a relatively robust simplification when there is a low amount of quadrivalent formation or preferential pairing. Of some concern are polyploid species for which the mode of inheritance is neither strictly polysomic nor disomic, but something in between. There have been various reports of “segmental allopolyploidy” (Stebbins 1947; Sybenga 1996), for example, in rose (Koning-Boucoiran et al. 2012), garden dahlia (Schie et al. 2014), and peanut (Leal-Bertioli et al. 2015). One of the advantages of our approach is that it predominantly relies on coupling-phase estimates which have been shown to be more robust against preferential pairing than repulsion-phase estimates [the case of S × N markers is covered in detail in (Bourke et al. 2015)]. We would caution against mapping in any polyploid species without first assessing the strength of preferential pairing, unless map construction is to be limited to a subset of marker segregation types (e.g., S × N and N × S with S × S or S × T markers, but not both). As mentioned, our mapping strategy can be tailored to accommodate differences in pairing behaviour between parents, chromosomes or even parts of a chromosome if necessary.

## Conclusions

In this study, we have demonstrated that high-quality, high-density linkage maps can be efficiently produced in tetraploid species, which we have applied to a data set from a biparental cross in the economically important crop species potato. These maps will facilitate downstream applications, such as QTL analysis and marker-assisted selection in polyploids. Our mapping approach results in the relatively fast creation of linkage maps in tetraploid species for which the assumption of random bivalent pairing holds to a reasonable extent. Extension to higher ploidy levels is theoretically straightforward, but remains to be realised in practice. Homologue mapping facilitates the parallelisation of map computation as well as providing long-range haplotype information, with marker phase being automatically assigned prior to mapping without the need for manual intervention. The time-limiting step remains marker ordering, but we have found that our binning approach offers a substantial speed-up in computational time without adversely affecting map quality.

### Author contribution statement

P.M.B. developed the methodology, performed the data analysis and wrote the manuscript. R.E.V. and C.M. conceived the study, helped develop the methodology and helped draft the manuscript. T.K. and J.J. helped develop the methodology and helped draft the manuscript. R.G.F.V. participated in coordination and helped draft the manuscript. All authors read and approved the final manuscript.

## Electronic supplementary material

Below is the link to the electronic supplementary material.

Supplementary material 1 (DOCX 114 kb)

Supplementary material 2 (XLSX 12 kb)

Supplementary material 3 (XLSX 522 kb)

Supplementary material 4 (XLSX 16 kb)

Supplementary material 5 (XLSX 897 kb)

Supplementary material 6 (R 10 kb)

Supplementary material 7 (XLSX 384 kb)

Supplementary material 8 (XLSX 387 kb)

Supplementary material 9 (XLSX 13 kb)

Supplementary material 10 (XLSX 13 kb)

Supplementary material 11 (XLSX 17 kb)

Supplementary material 12 (XLSX 16 kb)

Supplementary material 13 (PNG 183 kb)

Supplementary material 14 (PNG 310 kb)

Supplementary material 15 (PNG 323 kb)

Supplementary material 16 (PNG 225 kb)

Supplementary material 17 (PNG 245 kb)
