# Supplementary material for: Integrating haplotype-specific linkage maps in tetraploid species using SNP markers
Source: Theor Appl Genet. 2016 Aug 25;129(11):2211–26. doi: 10.1007/s00122-016-2768-1 (PMC5069339; doi:10.1007/s00122-016-2768-1)
Supplement: Supplementary file 1 — Supplementary material 1 (DOCX 114 kb) [file 122_2016_2768_MOESM1_ESM.docx]

# Appendix 1.

### Example derivation of the maximum likelihood equations for a SxT with a NxS marker.


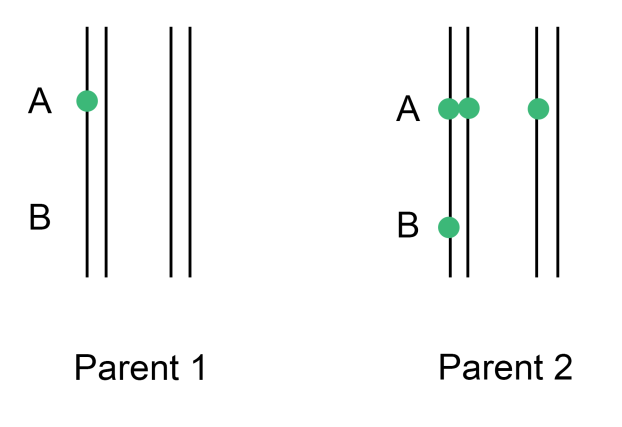


**Figure I.** Coupling pairing scenario 1

| **P1 gametes** | | **P2 gametes** | |
| --- | --- | --- | --- |
| aa/bb | ½ | Aa/bb | ¼ |
| Aa/bb | ½ | AA/bb | ¼ |
|  |  | Aa/Bb | ¼ |
|  |  | AA/Bb | ¼ |
| **F1 offspring** | | | |
| Aaaa/bbbb | | 1/8 | |
| AAaa/bbbb | | ¼ | |
| AAAa/bbbb | | 1/8 | |
| Aaaa/Bbbb | | 1/8 | |
| AAaa/Bbbb | | ¼ | |
| AAAa/Bbbb | | 1/8 | |

Both coupling and repulsion phases are possible although we don’t initially know which is correct and therefore must calculate both. If the reference allele at locus B is physically linked to one of the three reference alleles at locus A (in parent 2) we term the phase “coupling”. Under a random bivalent model, there are two possible pairing scenarios we must consider. In scenario 1, the homologue carrying the reference alleles for locus A and B is paired with another homologue carrying the allele for locus A (Figure I), which occurs with probability 2/3. If the recombination frequency between locus A and B is *r*, we can derive the possible gametes from P1 and P2 and their probabilities (where a small letter indicates the alternative allele). Combining these gives the possible offspring genotypes for this situation.

| **P1 gametes** | | **P2 gametes** | |
| --- | --- | --- | --- |
| aa/bb | ½ | Aa/bb | ½ (1-*r*) |
| Aa/bb | ½ | AA/bb | ½ *r* |
|  |  | Aa/Bb | ½ *r* |
|  |  | AA/Bb | ½ (1-*r*) |
| **F1 offspring** | | | |
| Aaaa/bbbb | | ¼ (1-*r*) | |
| AAaa/bbbb | | ¼ | |
| AAAa/bbbb | | ¼ *r* | |
| Aaaa/Bbbb | | ¼ *r* | |
| AAaa/Bbbb | | ¼ | |
| AAAa/Bbbb | | ¼ (1-*r*) | |

Scenario 2 pairing has probability 1/3 and is illustrated in Figure II. We again consider the possible gamete combinations and their probabilities (the probability of a non-recombinant is 1-*r*).

**Figure II.** Coupling pairing scenario 2


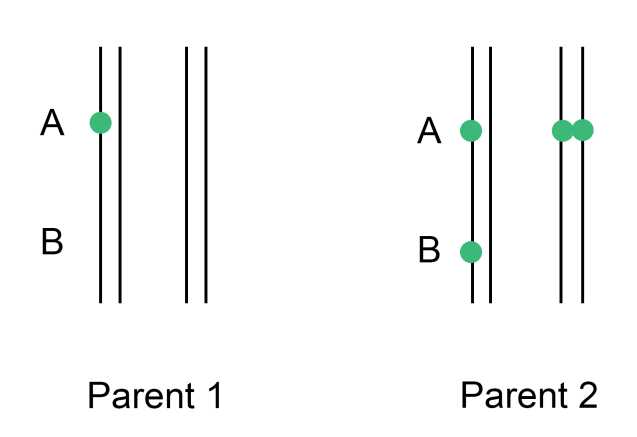


We now combine the probabilities from both pairing scenarios, scaled by their respective probabilities (2/3 and 1/3). These frequencies are then compared to the observed counts among the offspring in the six possible classes n_10_ to n_31_, where n_10_ refers to a single copy of the SNP allele at locus A and no copies of the SNP at locus B (as dosage scores) *etc*.

| **F1 offspring** | | |
| --- | --- | --- |
| Aaaa/bbbb | 1/12 (2-*r*) | n_10_ |
| AAaa/bbbb | ¼ | n_20_ |
| AAAa/bbbb | 1/12 (1+*r*) | n_30_ |
| Aaaa/Bbbb | 1/12 (1+*r*) | n_11_ |
| AAaa/Bbbb | ¼ | n_21_ |
| AAAa/Bbbb | 1/12 (2-*r*) | n_31_ |

The likelihood function follows; for details see *e.g.* (Van Ooijen and Jansen, 2013):

$$\mathcal{L}\left( r \right)=MC\left( \frac{1}{12}\left( 2-r \right) \right)^{n_{10}+n_{31}}{(\frac{1}{4})}^{n_{20}+n_{21}}\left( \frac{1}{12}(1+r) \right)^{n11+n30}$$

where *MC* refers to the multinomial coefficient, a constant which cancels in the subsequent calculations. The likelihood function (in fact, the log likelihood) is maximised to give the most likely estimate of *r* given the observed data ($\hat{r}$). This equation has an exact solution:

$$\hat{r}=\frac{2n_{11}-n_{10}-n_{31}+2n_{30}}{n_{10}+n_{11}+n_{30}+n_{31}}$$

In cases where neither marker has a SxN (or NxS) segregation type, the maximum likelihood equation cannot be solved analytically, and therefore a numerical maximisation method must be used (we used Brent’s algorithm (Brent, 1973)).

The LOD for linkage is defined as the log_10_ of the likelihood ratio, *i.e.*

$$\mathrm{LOD}=\log_{10} \left( \frac{\mathcal{L}\left( \hat{r} \right)}{\mathcal{L}\left( 0.5 \right)} \right)$$

The maximum likelihood framework for repulsion-phase follows a similar approach. To decide which phase is correct between the two markers, we select the phase for which the log likelihood is maximised (occasionally we select that for which *r* is minimised; see Results section “Optimal Phasing Strategy” for details.)

**“Integrating haplotype-specific linkage maps in tetraploid species using SNP markers”**, Theoretical and Applied Genetics, Peter M. Bourke, Roeland E. Voorrips, Twan Kranenburg, Johannes Jansen, Richard G. F. Visser, Chris Maliepaard. Wageningen UR Plant Breeding, Wageningen University and Research Centre, Droevendaalsesteeg 1, 6708 PB, Wageningen, The Netherlands. chris.maliepaard@wur.nl
