# Supplementary figures and images for: Integrating haplotype-specific linkage maps in tetraploid species using SNP markers
Source: Theor Appl Genet. 2016 Aug 25;129(11):2211–26. doi: 10.1007/s00122-016-2768-1 (PMC5069339; doi:10.1007/s00122-016-2768-1)

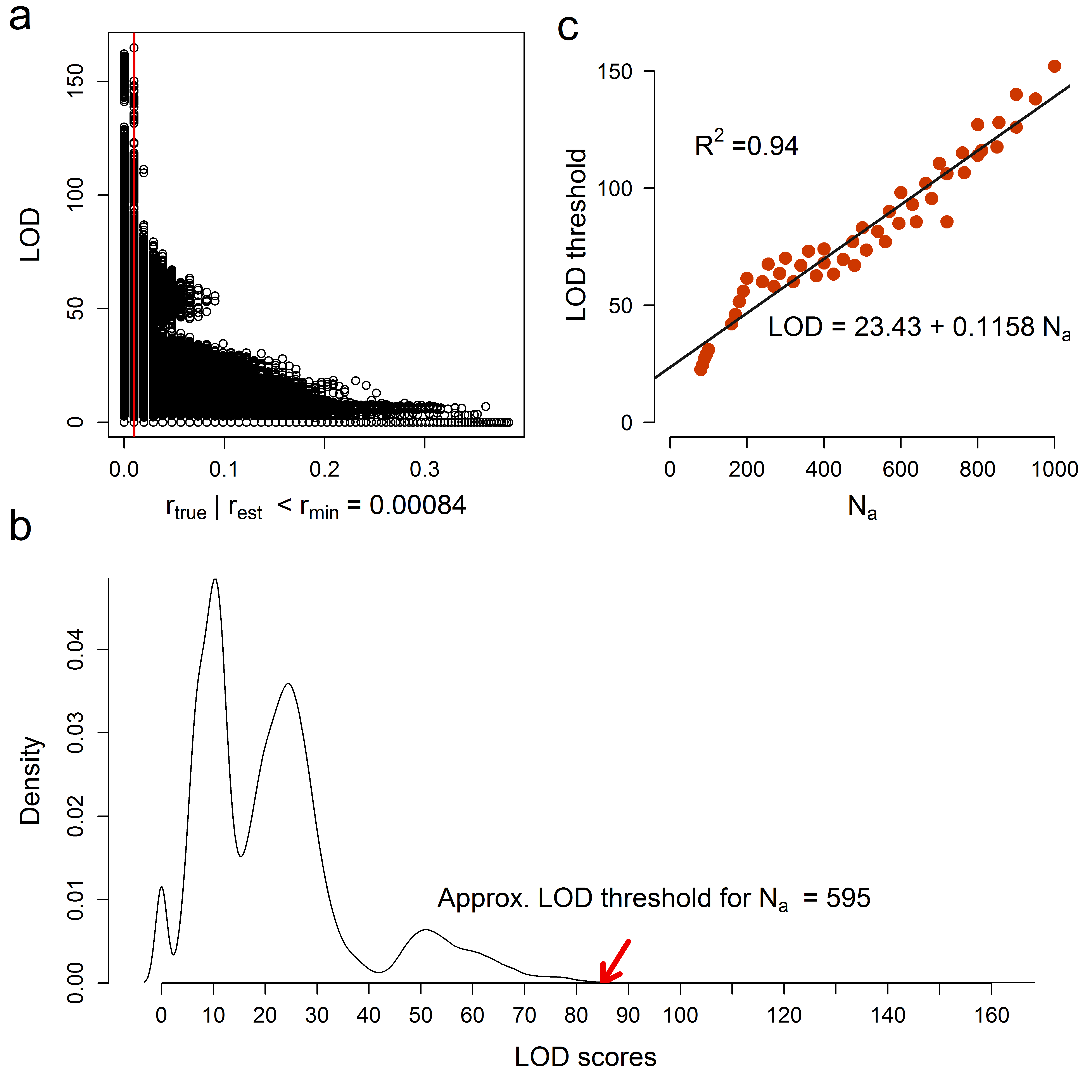

Supplement: Supplementary file 13 — Supplementary material 13 (PNG 183 kb) [file 122_2016_2768_MOESM13_ESM.png]

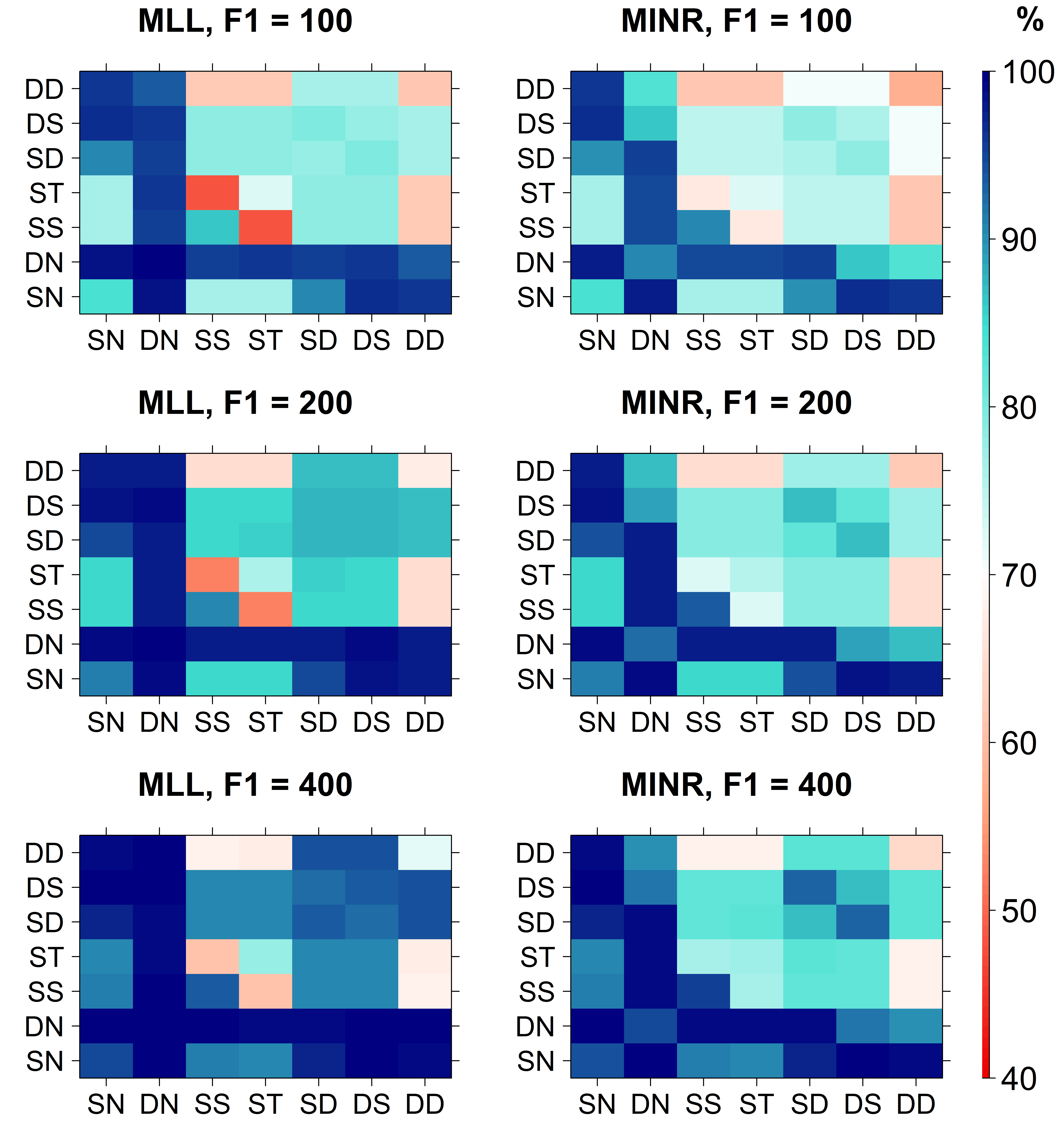

Supplement: Supplementary file 14 — Supplementary material 14 (PNG 310 kb) [file 122_2016_2768_MOESM14_ESM.png]

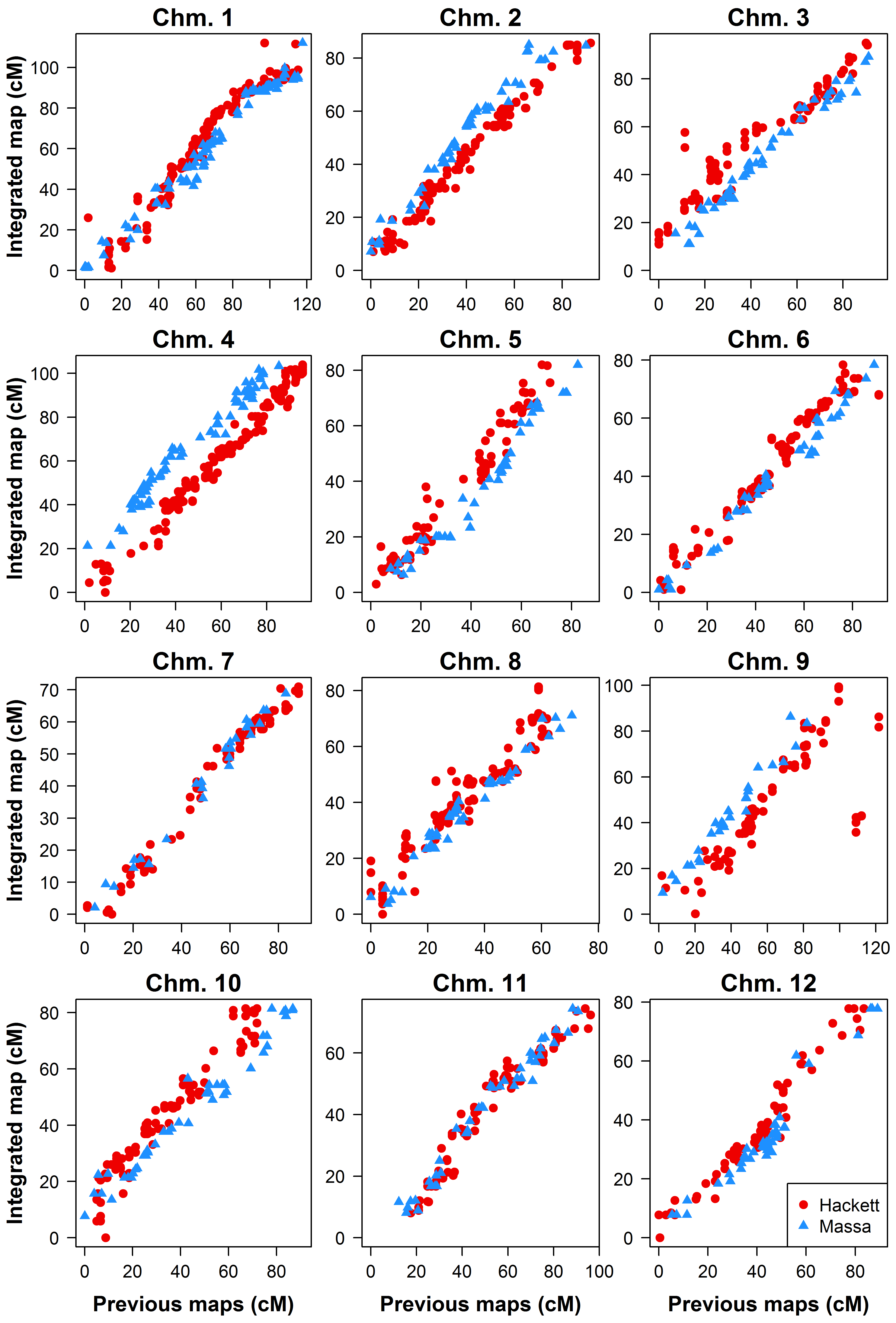

Supplement: Supplementary file 15 — Supplementary material 15 (PNG 323 kb) [file 122_2016_2768_MOESM15_ESM.png]

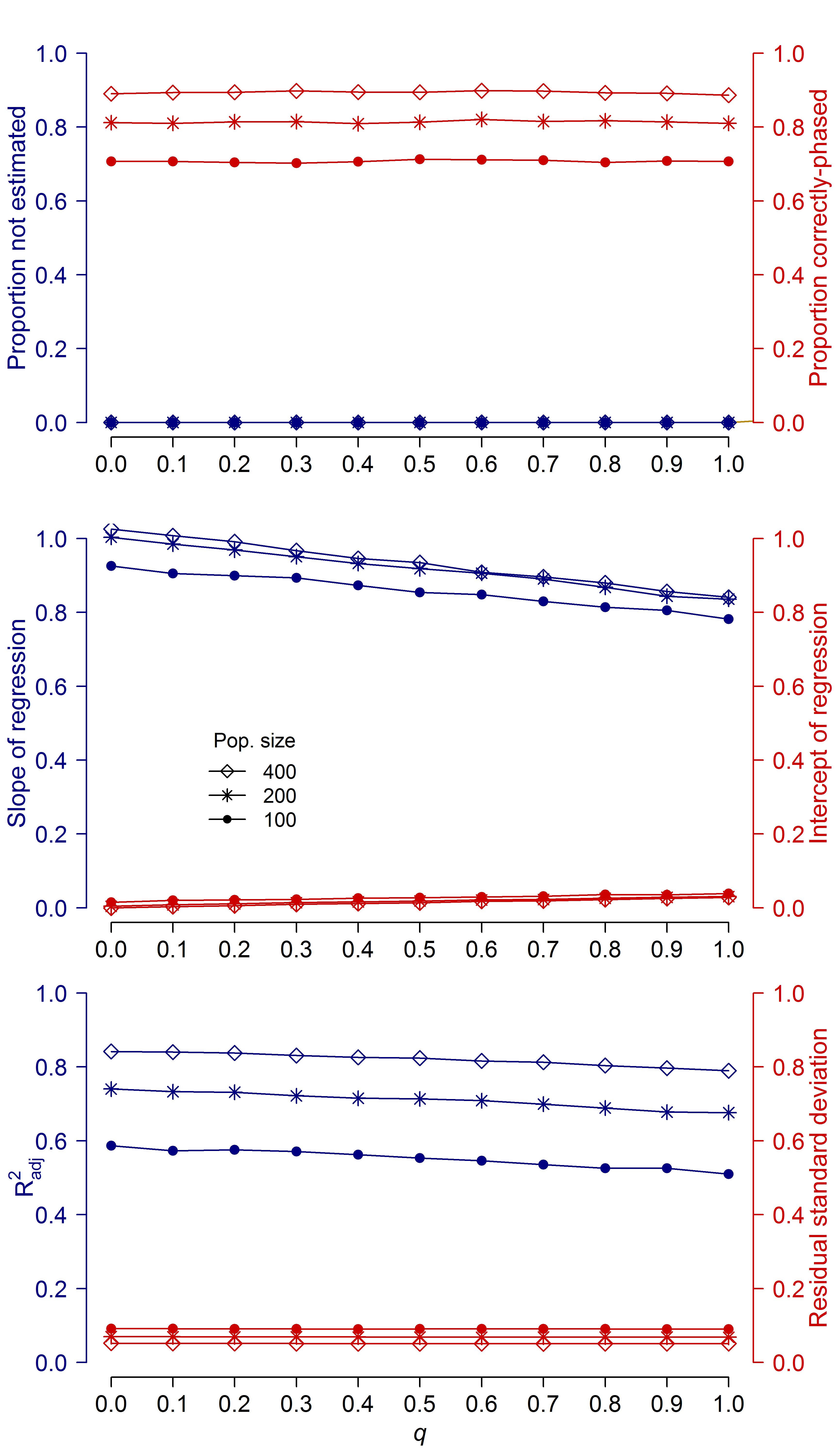

Supplement: Supplementary file 16 — Supplementary material 16 (PNG 225 kb) [file 122_2016_2768_MOESM16_ESM.png]

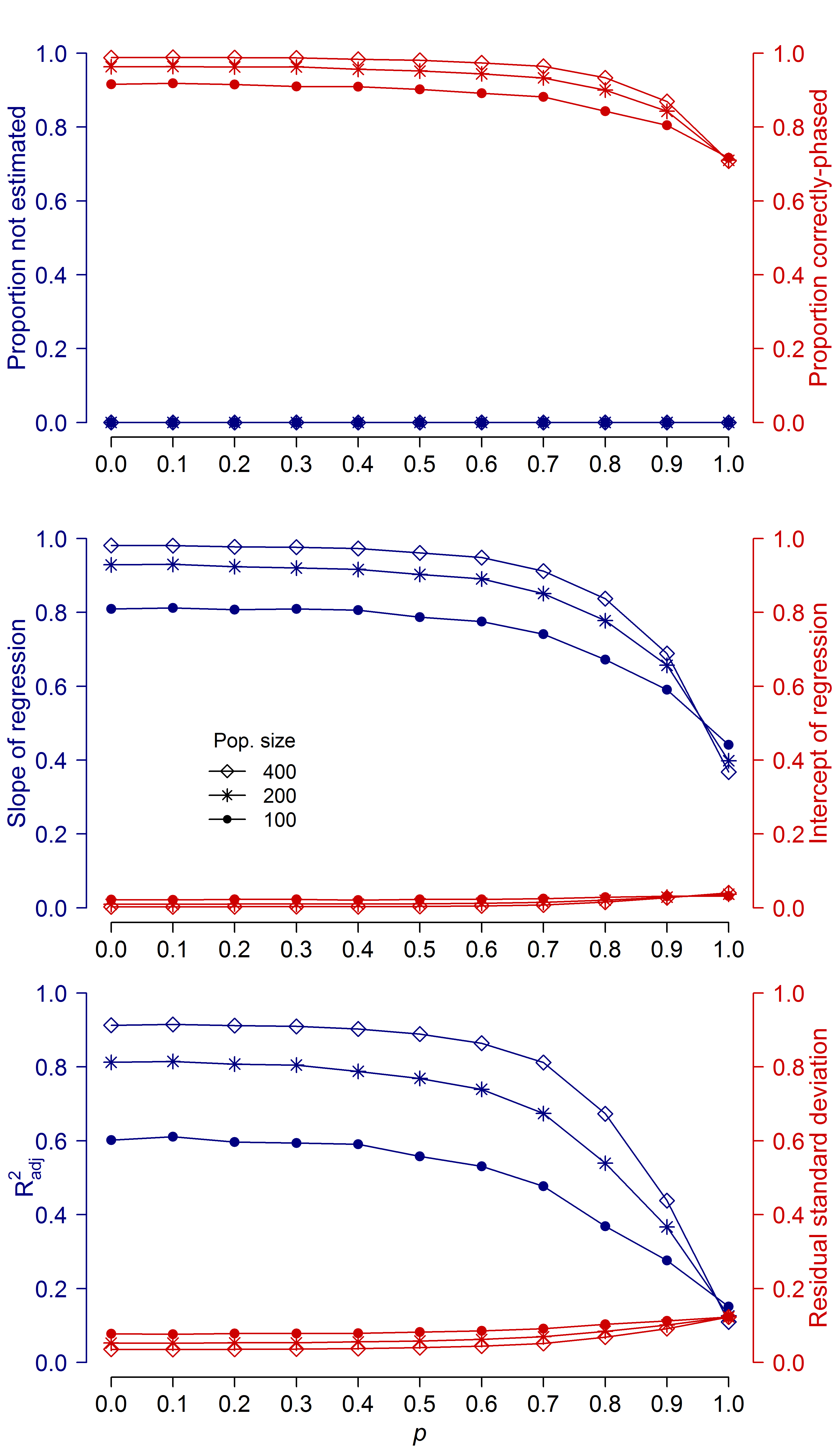

Supplement: Supplementary file 17 — Supplementary material 17 (PNG 245 kb) [file 122_2016_2768_MOESM17_ESM.png]
